# Voltammetry as a Tool for Characterization of CdTe Quantum Dots

**DOI:** 10.3390/ijms140713497

**Published:** 2013-06-27

**Authors:** Pavlina Sobrova, Marketa Ryvolova, Jaromir Hubalek, Vojtech Adam, Rene Kizek

**Affiliations:** 1 Department of Chemistry and Biochemistry, Faculty of Agronomy, Mendel University in Brno, Zemedelska 1, CZ-613 00 Brno, Czech Republic; E-Mails: pavlina.sobrova@seznam.cz (P.S.); Marketa.Ryvolova@seznam.cz (M.R.); vojtech.adam@mendelu.cz (V.A.); 2 Central European Institute of Technology, Brno University of Technology, Technicka 3058/10, CZ-616 00 Brno, Czech Republic; E-Mail: hubalek@feec.vutbr.cz; 3 Department of Microelectronics, Faculty of Electrical Engineering and Communication, Brno University of Technology, Technicka 10, CZ-616 00 Brno, Czech Republic

**Keywords:** quantum dot, electrochemistry, differential pulse voltammetry, cyclic voltammetry, *in vivo* imaging

## Abstract

Electrochemical detection of quantum dots (QDs) has already been used in numerous applications. However, QDs have not been well characterized using voltammetry, with respect to their characterization and quantification. Therefore, the main aim was to characterize CdTe QDs using cyclic and differential pulse voltammetry. The obtained peaks were identified and the detection limit (3 S/N) was estimated down to 100 fg/mL. Based on the convincing results, a new method for how to study stability and quantify the dots was suggested. Thus, the approach was further utilized for the testing of QDs stability.

## 1. Introduction

Quantum dots (QDs), semiconductor nanocrystals small enough to exhibit size-dependent properties, have generated tremendous interest due to their unique optical properties [1–6], including broad excitation spectra [7], narrow, tuneable [8] and symmetric emission spectra covering the wide range of spectra from visible to infrared, excellent photostability, and large quantum yield [9–14]. Their surface is also suitable for modification via incorporation of required functionality, and good biocompatibility [5,15–17], and they are also highly efficient multi-photon absorbers that can be potentially useful for three dimensional multi-photon microscopy and imaging [18]—a rapidly developing area for both biological and medical applications. These features make QDs one of the most promising nanomaterials for biological staining, detection of bio-macromolecules, and immunohistochemistry [16,19,20]. The most popular types of QDs include CdTe, CdSe, ZnSe, and ZnS; however, metals, such as In, Ga, and many others also can be used [21,22]. Despite the interesting properties hosted by these QDs, the potential leakage of metal ions by chemical dissolution under biological conditions may generate oxidative stress in living cells. Accordingly, the passivation of the surface of the QDs, in order to make them biologically inert without affecting their optical properties, becomes indispensable. Silicates, thiols, organic acids, peptides, proteins, and nucleic acids, forming bioconjugates have been used to cover the surface of QDs and, consequently, to reduce the possible leaking of free cadmium ions under physiological environments [16,23,24].

Electrochemical detection as a sensitive detection technique, in combination with QDs, has already been used for numerous applications such as immobilized substrate for glassy carbon electrodes [25,26] and/or modification of the surface of gold electrodes [27,28]. This powerful combination has also given rise to many electrochemical sensors for glucose [27], prostate specific antigen [29], thrombin [26], tumour cells [26], and/or allergens [30]. Electrochemical studies have also demonstrated several distinct oxidation and reduction peaks in the voltammograms with the peak positions being nanocrystal size dependent. It is demonstrated that the method is very sensitive to the nanocrystal surface states, providing complementary information for better understanding the optical properties of semiconductor nanocrystals [31]. However, to our knowledge, QDs have not been characterized well by voltammetry, with respect to use these easy-to-use and repeatable methods for QDs characterization and quantification. Therefore, the main aim was to characterize CdTe QDs using cyclic and differential pulse voltammetry. Based on convincing results, a new way to study stability, and quantify the dots, was suggested.

## 2. Results and Discussion

### 2.1. TEM Characterization of Synthesized Quantum Dots (QDs)

**Figure 1:**
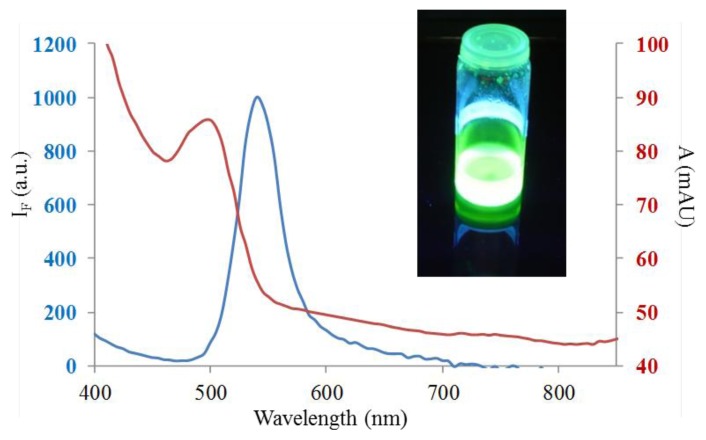
Emission (**blue line**) and absorption (**red line**) spectrum of CdTe quantum dots (QDs); inset: QDs solution under UV light illumination.

The TEM examination of prepared CdTe QDs indicated that the morphology and phase composition were clearly homogeneous. The transmission electron microscope (TEM) pictures (at higher magnifications) showed typical particle size was below 10 nm. Moreover, QDs solution under UV light illumination is shown in the left inset in Figure 1. Optical properties of synthesized QDs were further characterized spectrometrically. The absorption maximum at 500 nm was observed (Figure 1). From the emission spectrum showed in Figure 1 it is apparent that CdTe QDs are exhibiting strong fluorescence with the emission maximum at 525 nm. In the following parts of our experiments, we aimed our attention at their electrochemical characterization.

### 2.2. Voltammetry of QDs

Electrochemical techniques are known to show unique advantages in terms of both economic (relatively reasonable cost of equipment and very low operating costs) and excellent analytical features, *i.e.*, determination of low levels of metals in the buffer system and different matrix [32]. Compared to robust and highly expensive microscopic techniques like TEM, there is a need to have a method for rapid and low cost characterization and quantification of synthesized QDs. This was the motivation to study the electrochemical behaviour of QDs, which were composed of Cd and Te.

#### 2.2.1. Cyclic Voltammetry (CV) of QDs

**Figure 2:**
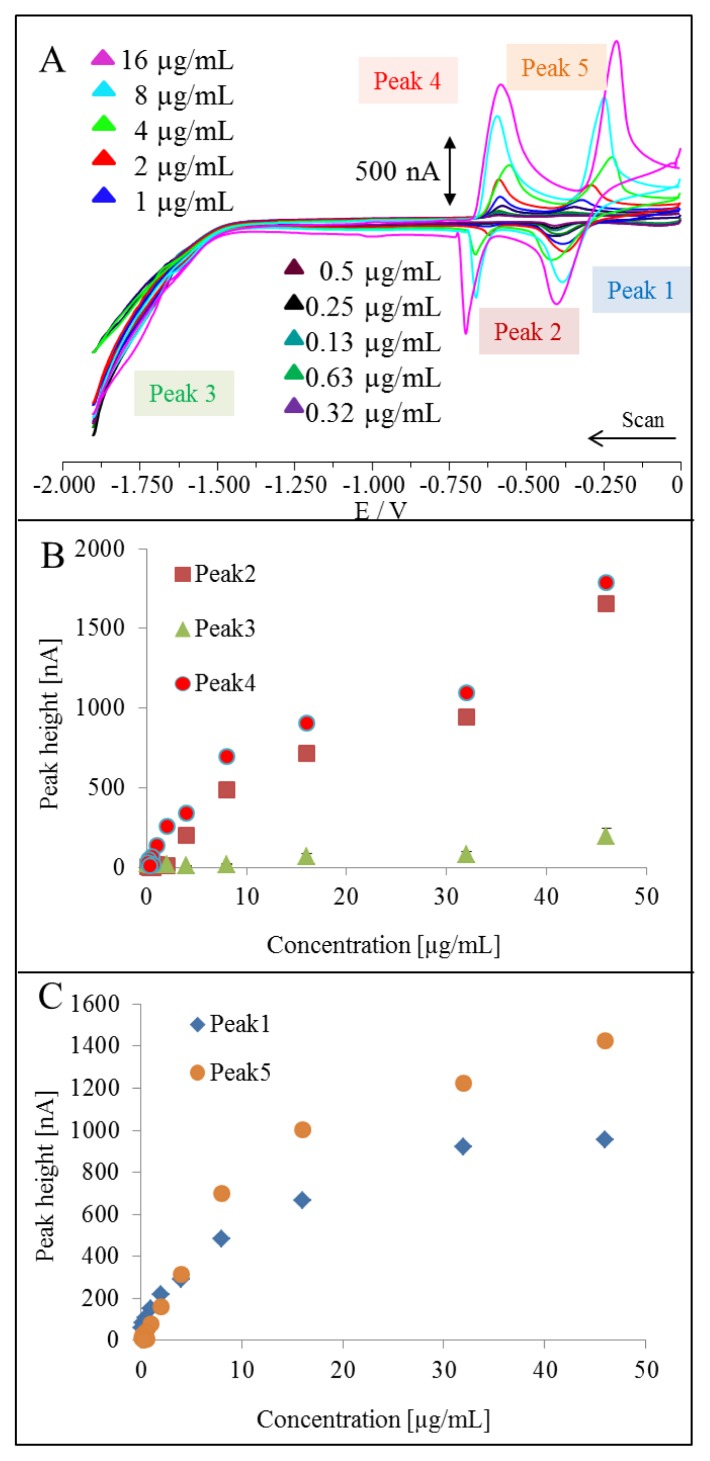
(**A**) Typical cyclic voltammograms of CdTe QDs measured at hanging mercury drop electrode (HMDE) *vs*. Ag/AgCl/3 M KCl; (**B**) Dependences of heights of peaks Nos. 2, 3, and 4 on concentration of QDs; (**C**) Dependences of the heights of peaks Nos. 1 and 5 on concentration of QDs. Acetate buffer (0.2 M, pH 5) was used as the supporting electrolyte. Cyclic voltammetry (CV) parameters were as follows: an initial potential 0 V, an end potential −1.9 V, vertex potential −1.5 mV, scan rate from 160 mV/s. All experiments were carried out at room temperature (22–24 °C). QDs were quantified according to results obtained by differential pulse voltammetry (DPV).

Cyclic voltammetry (CV) is perhaps the most widely used electrochemical technique, and is frequently used for the characterization of redox systems and is often the first experiment performed in an electroanalytical study. It can provide rapid information about the number of redox states of the electroactive species, as well as qualitative information about the stability of these oxidation states and the electron transfer kinetics. Based on the fact that chemicals used for synthesis of QDs are electroactive, we were interested in the issue what a cyclic voltammogram of QDs looked like. Typical cyclic voltammograms of CdTe QDs are shown in Figure 2A. Due to the presence of MPA, Cd, and Te, we obtained five peaks, which correspond to the redox reactions of the QDs. Peaks 1 and 5, and peaks 2 and 4, show reversible redox reactions of MPA and cadmium, respectively. Peak 3 could be of QD origin, but its height was several orders of magnitude lower when compared to the previously mentioned peaks (Figure 2B,C). These results indicate that QDs are electroactive, however, cyclic voltammetry is a method useful for redox characterization only. Quantification is doubtful due to poor sensitivity to QD signals.

#### 2.2.2. Differential Pulse Voltammetry (DPV) of MPA and Cadmium(II) Ions

**Figure 3:**
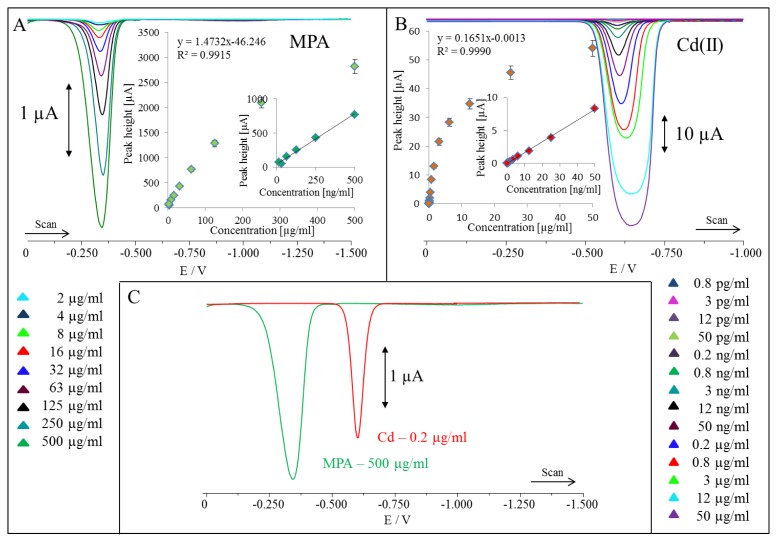
(**A**) Typical DP voltammograms of MPA measured at hanging mercury drop electrode (HMDE) *vs*. Ag/AgCl/3 M KCl; in inset: dependences of MPA peak height on its concentration; (**B**) Typical DP voltammograms of cadmium(II) ions measured at HMDE *vs*. Ag/AgCl/3 M KCl; in inset: dependences of Cd(II) peak height on its concentration; (**C**) Plotting of MPA and cadmium(II) ions peaks. Acetate buffer (0.2 M, pH 5) was used as the supporting electrolyte. Differential pulse voltammetric measurements were carried out under the following parameters: start potential −1.5 V; end potential 0 V; a modulation time 0.057 s, a time interval 0.2 s, a step potential of 1.05 mV/s, a modulation amplitude of 250 mV, *E*_ads_ = 0 V. All experiments were carried out at room temperature (22–24 °C). The DPV samples analyzed were deoxygenated prior to measurements by purging with argon (99.999%) saturated with water for 120 s.

Differential pulse voltammetry (DPV) as a method using fixed-magnitude pulses, which are superimposed on a linear potential ramp and applied to a working electrode at a time just before the end of the drop, is more sensitive to electroactive species compared to CV. DPV can be extremely sensitive for the determination of metal ions [33]. Therefore, we aimed our attention on the studying of the basic electrochemical behaviour of MPA and cadmium, as the components of our QDs of the highest content and highest redox activity as was shown in Figure 3A. MPA peaks at −0.350 V measured at hanging mercury drop electrode (HMDE) *vs*. Ag/AgCl/3 M KCl. The peak of MPA was proportional to its concentration as is shown in inset in Figure 3A. Detection limit of MPA (3 S/N) was estimated as 100 ng/mL. The same experiment was carried out for cadmium(II) ions (Figure 3B). These ions are well detectable at −0.600 V measured at HMDE *vs*. Ag/AgCl/3 M KCl. Peaks are well developed and also proportional to Cd(II) concentrations up to 50 μg/mL (inset in Figure 3B). After that, the increasing concentration of Cd(II) caused oversaturation of the electrode surface and the shape of the peak was not Gaussian, as is shown in Figure 3B. Moreover, the peaks were not proportional to the concentrations of QDs under concentration higher than 50 μg/mL. Due to extremely high sensitivity, we were able to detect 1 fg/mL (LOD, 3 S/N). In addition, we plotted both MPA and cadmium(II) ions peaks, which is shown in Figure 3C. In spite of the fact that we used higher concentrations of both chemicals, their peaks were well separated.

#### 2.2.3. Differential Pulse Voltammetry of QDs

**Figure 4:**
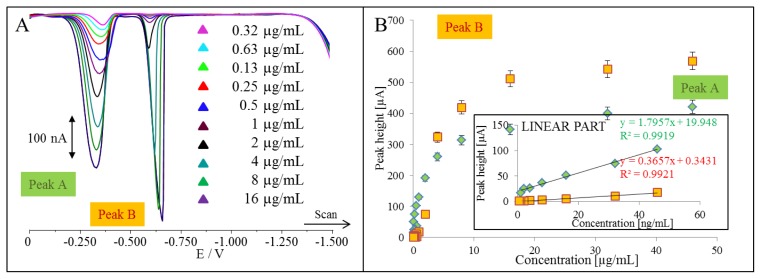
DP voltammetry of QDs. (**A**) Typical DP voltammograms of various concentrations of QDs; (**B**) Dependences of peaks A and B heights on the concentration of QDs. In insert: linear part of dependence of peak A and B heights on the concentration of QDs. All experimental parameters were the same as indicated in caption for Figure 3. QDs were quantified according to results obtained by DPV.

**Figure 5:**
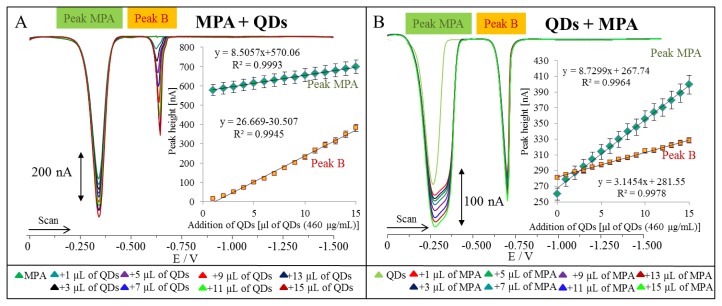
(**A**) DP voltammograms of MPA (500 μg/mL) with additions of QDs in various volumes (460 μg/mL, quantified according to concentration of Cd(II)); in inset: dependence of MPA and B peak heights on concentration of QDs; (**B**) DP voltammograms of QDs (460 μg/mL) with additions of MPA in various volumes (500 μg/mL); in inset: dependence of MPA and B peak heights on concentration of MPA.

Further, we aimed our attention differential pulse voltammetric characterization of QDs. Typical voltammograms of QDs are shown in Figure 4A. It clearly follows from the results obtained that both detected peaks called Peak A and Peak B enhanced with the increasing concentration of QDs (Figure 4B). The both dependences were of logarithmic shape. Therefore we split the curve and their linear parts are shown in inset in Figure 4B. To identify both peaks, we performed the experiments, in which we added MPA and/or cadmium(II) to solution containing QDs, and the mixture measured by DPV. We firstly focused on MPA. DP voltammograms of QDs additions (460 μg/mL, quantified according to concentration of cadmium(II)) to MPA are shown in Figure 5A. Based on the addition, we can conclude that peak A belongs to MPA. Both peaks, MPA and B, enhanced linearly with the increasing concentration of QDs (inset in Figure 5A). We performed this experiment in the opposite way, *i.e.*, additions of MPA (500 μg/mL) to QDs (Figure 5B). As we expected, we observed an increase in the peak of MPA, however, peak B was also enhancing with the increasing concentration of MPA (inset in Figure 5B). The increase was not sharp but considerable. This phenomenon can be related to influence of MPA additions on the nature of the peak B.

**Figure 6:**
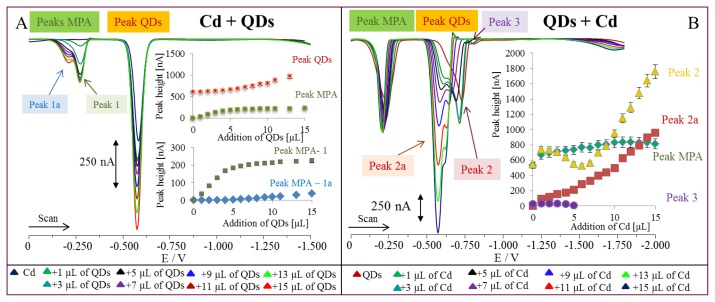
(**A**) DP voltammograms of Cd(II) (500 μg/mL) with additions of QDs in various volumes (460 μg/mL, quantified according to concentration of cadmium(II)); in inset: dependence of MPA (peak 1 and 1a) and QDs peak heights on concentration of QDs; (**B**) DP voltammograms of QDs (460 μg/mL) with additions of Cd(II) in various volumes (500 μg/mL); in inset: dependence of MPA and QDs peaks (peak 2, 2a, and 3) heights on concentration of Cd(II).

To find the nature of peak B, we tested the second electroactive substance, cadmium(II) ions, from which QDs were prepared. QDs (460 μg/mL) were added in the same concentrations as in the case of MPA (Figure 6A). Peak B renamed on peak QDs enhanced with the increasing concentration of the nanoparticles (inset in Figure 6A). Moreover, we found the dividing of MPA peak into Peak 1 and Peak 1a, which confirmed our hypothesis that MPA as well as cadmium(II) influenced their electroactivity. Double peaks should be related to excess of MPA, which could result in two peaks. The most interesting part was the testing of additions of cadmium(II) ions (500 μg/mL) to QDs. Based on the measured voltammograms, Peak MPA did not change, but peak QDs divided into three signals (Figure 6B). Peak 2a is of QDs origin, which was confirmed according to peak potential. The excess of cadmium(II) ions gave peak 2 and some MPA-QDs-Cd(II) complexes related to peak 3. All detected peaks enhanced with the increasing concentration of Cd(II) (inset in Figure 6B). After that, we found the nature of the detected peaks, we attempted to estimate detection limit for synthesized QDs. In order to estimate it, peak QDs was selected. Detection limit (3 S/N) was estimated as 100 fg/mL.

### 2.3. Stability of Quantum Dots

**Figure 7:**
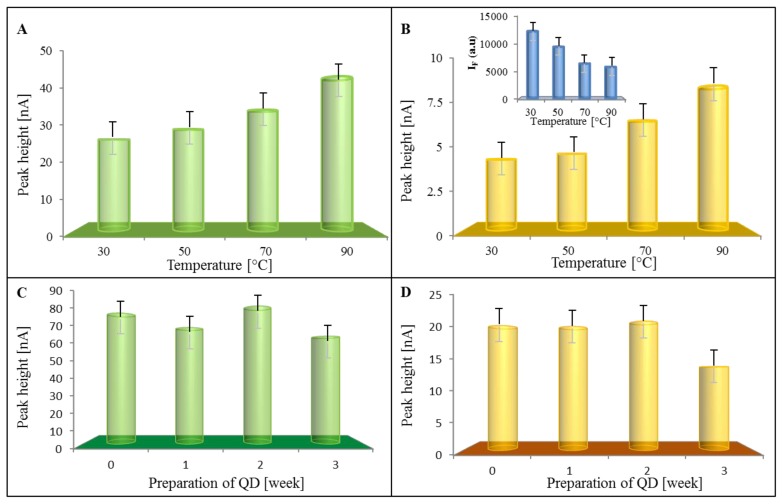
Dependences of the height of (**A**) MPA and (**B**) QDs peaks on temperature used for treatment of CdTe QDs; inset in (**B**): dependence of QDs emission on the temperature. The dots were heat treated for one hour. Dependences of the height of (**C**) MPA and (**D**) QDs peaks on time of storage of CdTe QDs.

The electrochemical studying of quantum dot behaviour gave a tool for their ultrasensitive quantification on one hand, but, based on the profile of detected peaks, there could be also possibility to monitor their stability on the other hand. Therefore, we focused on studying the time of stability of the used QDs. The decaying of QDs can be characterized by the detection of the decreasing of emission peak. We assume that this phenomenon can be also characterized by the increasing of two main electrochemical peaks called “MPA” and “QDs”. Primarily, temperature stability was investigated within the range from 30 to 90 °C. The dots were heat treated for one hour in the dark and then analyzed. The effects of temperature on peaks of MPA and QDs is shown in Figure 7A,B, respectively. It clearly follows, from the results obtained, that both peaks increased with the increasing temperature, which was associated with the decaying of the dots and was in good agreement with the emission decreasing (inset in Figure 7B). Based on these promising results, we followed with time stability tests. QDs were analyzed for three weeks. The height of MPA and QDs peaks are shown in Figure 7C,D, respectively. It can be concluded that the prepared dots are stable for two weeks without any changes, after which, some degradation occurred.

**Figure 8:**
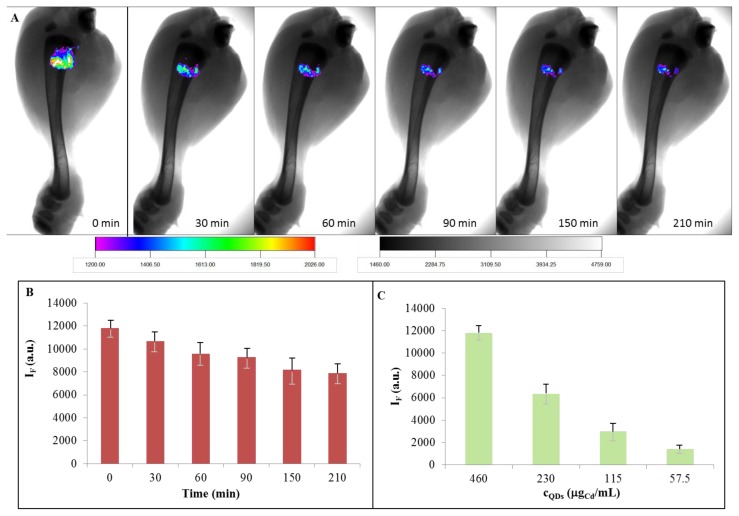
(**A**) *In vivo* images of 10 μL (460 μg/mL) of CdTe QDs applied 5 mm under the skin of the chicken thigh. The image of the chicken leg without any injection was subtracted; (**B**) The dependence of the emission of QDs on time after injection of the dots into a tissue; (**C**) The dependence of the emission of QDs injected into the chicken thigh on their concentration.

Further, we were interested in the issue whether our stable quantum dots could be used *in vivo*. Therefore, we investigated the fluorescence of QDs in an animal tissue. The chicken thigh was bought from Tesco store. The skin was removed from the chicken thigh and 10 μL (460 μg/mL) of QDs were injected into the muscle (Figure 8A). The intensity of the fluorescence decreased with the increasing time, as is shown in Figure 8B. It should be noted that the image of the chicken leg without any injection was taken first and was subtracted from all images to remove the autofluorescent background signal caused by tissues. Considering these facts, it is clear that the prepared QDs were stable enough to give considerable signal even after 210 min in a muscle. There is also need to quantify the intensity of the dots and to find the correlation between intensity and the concentration of the label. The dependence of QDs emission intensity on concentration of QDs injected into the chicken thigh is shown in Figure 8C. The intensity was determined immediately after application. The emission intensity decreased with decreasing concentration of QDs, proportionally.

## 3. Experimental Section

### 3.1. Chemicals

Cadmium chloride, sodium tellurite, mercaptopropionic acid, and other used chemicals were purchased from Sigma Aldrich (St. Louis, MO, USA). Stock solutions of 50 μg/mL of Cd(II), and 500 μg/mL of MPA were prepared daily and subsequently diluted to the appropriate concentration. Acetate buffer of pH 5 was prepared with 0.2 M acetic acid and 0.2 M sodium acetate, diluted with ACS water and used as a supporting electrolyte. High purity deionized water (Milli-Q Millipore 18.2 MΩ/cm, Bedford, MA, USA) was used throughout the study.

### 3.2. Microwave Assisted Preparation of Quantum Dots

QDs were prepared according to Duan *et al.* [34]. Cadmium chloride solution (CdCl_2_, 0.04 M, 4 mL) was diluted to 42 mL with ultrapure water, and then trisodium citrate dihydrate (100 mg), Na_2_TeO_3_ (0.01 M, 4 mL), MPA (119 mg), and NaBH_4_ (50 mg) were added successively under magnetic stirring. The molar ratio of Cd^2+^/MPA/Te was 1:7:0.25. 10 mL of the resulting CdTe precursor was put into a Teflon vessel. CdTe QDs were prepared at 95 °C for various times according to emission color (10 min—green; 30 min—yellow; 120 min—red) under microwave irradiation (400 W, Multiwave3000, Anton-Paar GmbH, Graz, Austria). After microwave irradiation, the mixture was cooled 50 °C and the CdTe QDs sample was obtained. Repurification of CdTe QDs was carried out using isopropanol condensing. The CdTe QDs were mixed with isopropanol in ratio 1:2 and then centrifuged 10 min at 25,000 rpm (Eppendorf centrifuge 5417R, Hamburg, Germany). Supernatant (pure CdTe QDs) was then resuspended in the initial volume of Tris Buffer pH 8.5.

### 3.3. Electrochemical Analyzer

Electrochemical measurements were performed with an AUTOLAB Analyzer (EcoChemie, Utrecht, The Netherlands) connected to VA-Stand 663 (Metrohm, Herisau, Switzerland), using a standard cell with three electrodes. The working electrode was a hanging mercury drop electrode (HMDE) with a drop area of 0.4 mm^2^. The reference electrode was an Ag/AgCl/3 M KCl electrode and the auxiliary electrode was a graphite electrode. Acetate buffer (0.2 M, pH 5) was used as the supporting electrolyte. For smoothing and baseline correction GPES 4.9 software, supplied by EcoChemie, was employed.

#### 3.3.1. Cyclic Voltammetry (CV)

QDs were studied using cyclic voltammetry. The amount of measured sample was 2 mL in an electrochemical cell. CV parameters were as follows: an initial potential 0 V, an end potential −1.9 V, vertex potential −1.5 mV, scan rate from 160 mV/s. All experiments were carried out at room temperature (20 °C).

#### 3.3.2. Differential Pulse Voltammetry (DPV)

The amount of QDs was measured using DPV. Differential pulse voltammetric measurements were carried out under the following parameters: start potential −1.5 V; end potential 0 V; a modulation time 0.057 s, a time interval 0.2 s, a step potential of 1.05 mV/s, a modulation amplitude of 250 mV, *E*_ads_ = 0 V. All experiments were carried out at room temperature (20 °C). The DPV samples analyzed were deoxygenated prior to measurements by purging with argon (99.999%) saturated with water for 120 s.

### 3.4. Transmission Electron Microscope

Morphology studies and phase analysis were carried out with a transmission electron microscope (TEM) Philips CM 12 (tungsten cathode, using a 120 kV electron beam, Eindhoven, The Netherlands). Chemical compositions were studied by energy-dispersive X-ray spectroscopy (EDX, JEOL, Akishima, Japan). Electron diffraction patterns were simulated with JEMS software. Samples for TEM measurements were prepared by placing drops of the solution (sample and water) on coated Cu grids (holey carbon and holey SiO_2_/SiO) and subsequently drying in air.

### 3.5. Spectroscopy

Absorbance and fluorescence spectra were acquired by a multifunctional microplate reader Tecan Infinite 200 PRO (TECAN, Männedorf, Switzerland). The sample (50 μL) was placed in a transparent 96 well microplate with a flat bottom (Nunc, Thermo Scientific, Freiburg, Germany). Absorbance scan was measured in the range from 230 to 1000 nm using 5 nm steps. Three hundred and fifty nanometers was used as an excitation wavelength, and the fluorescence scan was within the range from 400 to 850 nm (5 nm steps). The detector gain was set to 50. For both absorbance and fluorescence measurements, each value was an average of five measurements.

### 3.6. *In vivo* Imaging

The fluorescence imaging was carried out by *In vivo* Xtreme system from Carestream, USA. This instrument is equipped with a 400 W xenon light source and 28 excitation filters (410–760 nm). The emitted light is captured by a 4MP CCD camera. In this experiment, 410 nm was used as an excitation wavelength and the emission was measured at 535 nm. The exposition time was 5 s. The other parameters were set as follows: Bin—2 × 2, field of view—12 cm, *f*_Stop_—1.1.

The X-ray image was taken as a background image using following setting: exposure time—1.2 s, filter—0.2 mm, Bin—1 × 1, power—45 KVP. Between each measurement, the tray with the chicken leg was kept at 4 °C.

### 3.7. Descriptive Statistics and Estimation of Detection Limit

Data were processed using MICROSOFT EXCEL^®^ (Redmond, WA, USA). Results are expressed as mean ± standard deviation (S.D.) unless noted otherwise (EXCEL^®^). The detection limits (LOD, 3 signal/noise, S/N) were calculated according to Long and Winefordner [35], whereas N was expressed as standard deviation of noise determined in the signal domain unless stated otherwise.

## 4. Conclusions

There are several methods, which can be used for characterization of quantum dots. These methods are often laborious and instruments have high costs. Our results suggest that both cyclic and differential pulse voltammetry can be considered as extremely sensitive, easy-to-use, and a low cost method for the rapid characterization of quantum dots. With LOD down to fg per mL, quantum dots should be of interest also as an electroactive label. Moreover, we showed that QDs were stable enough to be applied into a muscle.
